# Sedentary Time, Physical Activity Levels and Physical Fitness in Adults with Intellectual Disabilities

**DOI:** 10.3390/ijerph18095033

**Published:** 2021-05-10

**Authors:** Po-Jen Hsu, Hung-Shih Chou, Yi-Hsiang Pan, Yan-Ying Ju, Chia-Liang Tsai, Chien-Yu Pan

**Affiliations:** 1 Graduate Institute of Physical Education, National Taiwan Sport University, Taoyuan 333, Taiwan; a0912024720@gmail.com (P.-J.H.); hschou@ntsu.edu.tw (H.-S.C.); a0922302951@gmail.com (Y.-H.P.); 2 Department of Adapted Physical Education, National Taiwan Sport University, Taoyuan 333, Taiwan; yanju@365.ntsu.edu.tw; 3 Institute of Physical Education, Health and Leisure Studies, National Cheng Kung University, Tainan 701, Taiwan; andytsai@mail.ncku.edu.tw; 4 Department of Physical Education, National Kaohsiung Normal University, Kaohsiung 802, Taiwan

**Keywords:** physical fitness, intellectual disability, physical activity, sedentary time, accelerometer

## Abstract

*Purpose*: This cross-sectional study assessed the associations of gender, age, level of intellectual disabilities (IDs) and of daily sedentary and physical activity (PA) time with physical fitness in adults with ID. *Materials and methods*: Sixty adults (mean age = 39.19 ± 11.70 years) with ID participated in this cross-sectional study. PA was monitored for 7 days using an ActiGraph GT3X monitor. Physical fitness was measured with a 6-min walking test, isometric push-up test, modified curl-up test, handgrip strength test, and back-saver sit-and-reach test. *Results*: (a) An age of ≥39 years and female gender were associated with lower performance in multiple aspects of physical fitness. (b) More moderate-to-vigorous PA (MVPA) was associated with greater muscular strength and endurance (modified curl-ups: β = 0.36, *p* < 0.01; handgrip strength: right, β = 0.52, *p* < 0.01; left, β = 0.52, *p* < 0.01). (c) More light-intensity PA (LPA) was associated with greater upper-body muscular endurance (β = 0.42, *p* < 0.01) and greater flexibility (right leg: β = 0.36, *p* < 0.01; left leg: β = 0.38, *p* < 0.01). *Conclusion*: LPA may be as beneficial as MVPA to the physical fitness of adults with ID. Future studies should focus on developing effective PA interventions for adults with ID, especially for women and individuals aged ≥39 years, by incorporating both LPA and MVPA.

## 1. Introduction

People with intellectual disability (ID) may exhibit limited adaptive capacity in not only their mental development but also their physiological, social, and emotional development. Furthermore, they may experience more health problems than the general population does, including epilepsy, visual and hearing impairment, gastrointestinal problems, dementia [1] and sensorimotor dysfunction [2]. Dysfunctions of the central nervous system in individuals with ID cause movement difficulties as well as problems with coordination and the proprioception. Consequently, people with ID are less physically active [3], live a more sedentary lifestyle [4], have lower levels of physical fitness [5], and are more likely to be overweight or obese [6] than the general population is. Such low physical activity (PA) and frequent sedentary behavior are associated with negative health outcomes reflective of the health inequalities faced by adults with ID, including reduced life expectancy [7] and an increased prevalence of coronary heart disease [8]. The majority of research suggests that these individuals engage in insufficient PA [9,10]; however, information on how ID is reflected in PA levels and various fitness components is lacking but required for developing targeted PA and fitness interventions for individuals with ID.

Most definitions of physical fitness incorporate body composition, cardiorespiratory endurance, muscular strength and endurance, and flexibility [11]. These physical aspects are essential for performing activities of daily living and developing functional skills, and low levels of cardiorespiratory fitness and muscular strength and endurance can limit independence in adulthood [12]. Poor physical fitness is a risk factor for cardiovascular diseases, diabetes mellitus, and poor mental health [13]. Several longitudinal studies have reported that risk factors of cardiovascular diseases, such as hypercholesterolemia, hypertension, and overweight, persist from childhood into adulthood [14,15,16]. Adults with ID have been demonstrated to be less physically fit than the general population [5,17]. The prevention and treatment of poor physical fitness are critical for limiting care dependency and costs and for ensuring individuals with ID have the skills required for maintaining health and well-being while aging [18,19]. To develop physical fitness programs and health policies for adults with ID, the determinants of physical fitness in such adults must be discovered.

Gender, age, and ID level have been identified as correlates of physical fitness for adults with ID [5,20,21]; however, whether such correlations are significant in this population remains inconclusive. Hilgenkamp et al. [20] assessed the relationships of multiple individual factors (e.g., age, gender, and ID level) with physical fitness in people with ID aged 50 years and over. They [20] indicated that being older, being female, and having more severe ID were independently associated with lower levels of multiple physical fitness parameters. Specifically, women had slower walking speeds (at both a comfortable and fast pace) and weaker grip strength than men but had greater flexibility. Older age was associated only with slow walking speed. More severe ID was negatively associated with performance on 8 out of 11 physical fitness tests—all except those testing walking speed, percentage of maximal heart rate achieved, and flexibility. Gawlik et al. [5] assessed the aerobic capacity of adults with ID aged 20–40 years and its relationships with age, gender, ID level, and somatic variables; age and ID level were not significantly associated with physical fitness. The absolute magnitudes of physical work capacity at a heart rate of 170 beats/min and VO_2max_ were significantly higher in men; however, aerobic capacity expressed proportionally to body weight did not differ between genders. Somatic parameters, including weight, waist and hip circumference, body fat percentage, body mass index (BMI), and waist-to-hip ratio, were discovered to be differentiating factors of aerobic capacity in the study population. Cuesta-Vargas et al. [21] examined the physical fitness profiles of adults with mild ID (*N* = 266; mean age = 31.1 ± 7.5 years) and discovered that women scored higher than men only on flexibility tests. Overall, the data on this topic seem insufficient for identifying and explaining a link between physical fitness and fixed individual characteristics in adults with ID.

In addition to fixed individual characteristics, a modifiable variable that can be targeted in rehabilitation and interventions is PA. Regular PA has comprehensive and complex effects on the ability to adapt and perform activities of daily living as well as on personal, social, and professional function in the general population. However, information on the relationship between PA and fitness in adults with ID is scarce and inconclusive. Hilgenkamp et al. [20] assessed the relationships between PA and fitness in older adults with ID. They measured PA with a pedometer and classified participants into an active group (≥7500 steps/day) and a less active group (<7500 steps/day). Participants with high PA scored higher on tests of balance, comfortable and quick walking speed, muscular endurance of the legs, and cardiorespiratory endurance. Walsh et al. [22] compared the PA, fitness levels, BMI, and blood pressure of adults with ID (33.01 ± 11.09 years) between participants and nonparticipants in Special Olympics programs. Those who participated accumulated more moderate-to-vigorous PA (MVPA) per day and had higher fitness levels and health profile scores than did those who did not participate in the programs. By contrast, Cuesta-Vargas et al. [21] investigated differences in physical fitness performance between highly physically active (PA for 3–7 h/week; *n* = 118) and less active adults with mild ID (1–2 h/week; *n* = 148) aged 31.10 ± 7.50 years and discovered that the two groups demonstrated similar fitness levels. The authors [21] suggested that further studies should be undertaken to more accurately determine the level of PA and fitness profiles of individuals with ID; practical applications of such studies could include community programs appropriate for improving the health of this population.

Current PA guidelines recommend that adults aged 18 to 64 years, including those with disabilities, engage in at least 150 min of moderate-intensity or 75 min of vigorous-intensity aerobic activity (or an equivalent combination in bouts of ≥10 min) each week to achieve health benefits [23]. When unable to meet these guidelines, adults with disabilities should engage in regular PA according to their specific health risks and limitations and avoid a sedentary lifestyle whenever possible. The benefits of MVPA for the prevention of major non-communicable diseases, including type 2 diabetes, coronary heart disease, stroke, and some cancers [24], are well known, but evidence of the health benefits of light-intensity PA (LPA) is growing. In a systematic review, Amagasa et al. [25] analyzed the associations of objectively measured LPA with various health outcomes after adjustment for MVPA in adults and older adults and revealed that objectively measured LPA was inversely associated with the risk of all-cause mortality and favorably associated with some cardiometabolic risk factors, including waist circumference, triglyceride levels, insulin levels, and metabolic syndrome. Although LPA has great potential to increase overall PA, the precise effects of LPA remain uncertain. With advances in objective PA monitoring technology, the associations of daily PA and PA intensity with physical fitness can be explored more effectively [26,27].

Therefore, the present study assessed the associations of physical fitness components with (a) the gender, age, and ID level and (b) sedentary activity (SA) duration and PA levels in adults with ID. Understanding the associations of these factors with physical fitness is crucial for informing the development of intervention programs aimed at reducing or preventing obesity and promoting active lifestyles from early to late adulthood. The authors hypothesized that various fitness components would be positively and negatively associated with PA and SA, respectively.

## 2. Methods

### 2.1. Participants

This cross-sectional study recruited individuals with ID from two disability service provider organizations in two large cities in Taiwan. Members of the research team met with a staff liaison from each organization and explained the study’s purpose and processes. All individuals with ID who were registered with the service providers were invited to participate. The inclusion criteria were (a) age of 18 years or over, (b) mild or moderate ID, and (c) sufficient verbal communication skills and physical capacity to follow instructions and complete all the tests independently. The exclusion criteria were any contraindication to exercise and any medication that may affect activities of daily living. In total, 60 individuals with ID (27 women and 33 men) were included.

The participants were all on the Taiwan nationwide registry of people with disabilities. Generally, ID classification is based on a combination of IQ and adaptive behavior, and ID is categorized into one of four classes by a physician: mild, moderate, severe, or profound. The medical diagnoses of mild and moderate ID in this study were, respectively, based on the International Classification of Diseases, 10th Revision, Clinical Modification codes F70 and F71.

### 2.2. Procedure

This study was approved by the Institutional Research Ethics Committee for the Protection of Human Subjects of National Cheng Kung University (HREC-F-109-032-2). The informed consent form was signed by the participant and co-signed by a parent or guardian after an explanation of the research study and what would be expected of them. Height and weight measures were collected in a private setting with all participants dressed in light clothing and shoes removed for calculation of BMI (kg/m^2^). All physical fitness testing was performed in a quiet and isolated activity room at the disability service providers’ locations immediately before or after the 7 days of PA monitoring. All participants were accompanied by a service worker from the organization or a family member during data collection.

### 2.3. Anthropometric Measurements

Height was measured to the nearest 0.1 cm using a stadiometer (Seca 225, Seca, Hamburg, Germany). Weight was measured to the nearest 0.1 kg on a digital scale (Seca 861, Seca) with the participants wearing lightweight clothing and no shoes. BMI was calculated as weight in kilograms divided by height in meters squared (kg/m^2^). BMI was categorized according to the World Health Organization’s classification [28]: <18.49 kg/m^2^ is underweight, 18.5–24.99 kg/m^2^ is normal weight, 25–29.99 kg/m^2^ is overweight, and ≥30 kg/m^2^ is obese.

### 2.4. Physical Activity (PA) Measurements

PA was monitored and objectively recorded using ActiGraph accelerometers (GT3X, Firmware 4.4.0, ActiGraph, Fort Walton Beach, FL, USA). The accelerometers were programmed to measure activity in 60 s epochs. The participants and their caregivers or legal guardians were provided with instructions on how to wear the accelerometer; they were asked to wear the device over their right hip on an elastic belt for 7 days when awake and to take it off while bathing, showering, or swimming. The accelerometers were collected by the study investigators at disability service providers’ locations. Raw accelerometer counts were downloaded using ActiLife software (version 6.12.0) and used to calculate the time spent on SA, LPA, and MVPA as well as total PA measured in steps per day.

For inclusion in the analysis, each participant needed a minimum of 4 valid days of data, defined as 10 h or more of wear time. Non-wear time was defined as any interval of at least 60 min of zero activity counts, with an allowance for up to 2 min of 1 to 100 counts/min [29]. To categorize time spent (min/day) in the different PA intensities, SA (0 to 99 counts/min), LPA (100 to 1951 counts/min), and MVPA (≥1952 counts/min), accelerometer count cut points were calculated using Freedson cut-points for adults [30]. To obtain the average time per day spent on each level of PA, the total time (min) for that intensity level was divided by the number of days for each participant.

### 2.5. Physical Fitness Parameter Measurements

Physical fitness was measured with a range of instruments and a standard testing procedure [31,32,33]. All the participants completed tests measuring the following fitness components: (a) Cardiorespiratory fitness functioning was measured with the 6-minute walking test (6MWT). (b) Upper-body muscular endurance was assessed with an isometric push-up test. (c) Abdominal muscular endurance was measured with a modified curl-up test. (d) Hand and forearm muscular strength were assessed with a handgrip strength test. (e) Lower-body flexibility was measured with a back-saver sit-and-reach test. These fitness testing protocols are chosen because they are easier to administer and have been used often in clinical settings for adults as well as children and adolescents with ID [22,32,34,35]. The raw scores were used for data analysis.

#### 2.5.1. Cardiorespiratory Fitness

The 6MWT assesses functional capacity and is recommended by the American College of Sports Medicine as a field-based test of cardiorespiratory fitness for several populations with low fitness levels [36]. The test is a reliable and valid tool for measuring functional exercise capacity in individuals with ID [35,37,38]. In the original 6MWT, each participant independently walks 30 m back and forth down a corridor to cover the greatest distance possible in 6 min, with standardized encouragement [39]. The test was modified to make it more suitable for participants with ID [33]. In the modified 6MWT, the participants walk in a 20 m square and are encouraged individually to keep walking as quickly as they can. The total distance covered in 6 min is recorded in meters.

#### 2.5.2. Upper-Body Muscular Strength and Endurance

Upper-body muscular strength and endurance was measured using the isometric push-up test [32], which required the participants to attempt to sustain a raised plank position with their hands directly below their shoulders, arms fully extended, whole body in a straight line, and toes touching the floor for as long as possible up to 40 s. Scoring was terminated after 40 s or when the correct position could not be maintained. The time was recorded to the nearest 0.1 s.

#### 2.5.3. Abdominal Muscular Strength and Endurance

Abdominal muscular strength and endurance was measured using a modified curl-up test [32], in which the participants lay in a supine position on a mat with knees bent at approximately 140° and feet placed flat on the floor, legs held slightly apart, and hands placed on the front of the thighs. During the test, the participants had to contract their abdominal musculature, slide their hands along the thighs until the fingertips contact the patellae and then returned to the starting position. The fingers had to slide at least 10 cm to the patellae, and fingers were not allowed to lift off the legs and the hands had to slide up simultaneously to the left and right knee caps, respectively. The tester can place their hands on the participant’s knee cap to assist the participant in performing the correct movement. Participants completed as many curl ups as possible at a rate of one curl every 3 s, and the tester verbally counted the number of curl-ups. The test was ended at 75 curl-ups or when the participant was no longer able to maintain the pace of curl-ups by using the appropriate form. One trial was administered and the total number of curl-ups performed correctly was recorded.

#### 2.5.4. Hand and Forearm Muscular Strength

The handgrip test is a standardized method for assessing strength of the hand and forearm muscles, and scores on the test have been correlated with upper-extremity function [32]. The test involves gripping an adjustable handgrip dynamometer three times with each hand; the dynamometer is used to measure, and the middle score from the three trials is recorded. The participant must keep their arm and hand at their side with their elbow bent at 90° while squeezing as forcefully as possible. Handgrip dynamometers are highly reliable and valid (intraclass correlation coefficient = 0.98 and 0.99, respectively) for measuring handgrip strength [40].

#### 2.5.5. Flexibility

Flexibility was measured using the back-saver sit-and-reach test [32], which, in this study, required participants to reach the most distant point on a ruler with their fingertips. Participants removed their shoes and sat with one leg straight and the other bent in a sit-and-reach apparatus (30 cm high × 30 cm wide), keeping the foot of the bent leg next to the knee of the straight leg. Their arms were extended over the measuring scale with their palms facing downward and hands placed one on top of the other. The participants reached directly forward with both hands along the scale toward the sit-and-reach box and must hold the final reach position for at least 1 s; each participant performed four reaches. The participants then switched legs, and performed a second set of four reaches. One trial (4 stretches, holding the last) is given to each leg, and the distance was recorded in centimeters.

### 2.6. Statistical Analysis

Means (*M*) and standard deviations (*SD*) are provided for all variables. The nonparametric Mann–Whitney U tests were used to compare each fitness variable between groups of different genders, ages, and ID levels. The partial correlations—controlled for gender, age, and ID level—of physical fitness components with SA time and each PA parameter were calculated for the whole sample. The correlations were classified as done previously [41]: (a) 0.0 to 0.25 = little correlation, (b) 0.26 to 0.49 = low correlation, (c) 0.50 to 0.69 = moderate correlation, (d) 0.70 to 0.89 = high correlation, and (e) 0.90 to 1 = very high correlation. Stepwise multiple regression was used to determine the variance of PA variables significantly associated with physical fitness. The chosen PA variables were included in the multiple regression analysis to compare their strength in predicting each physical fitness component. The dependent variables were body composition (i.e., BMI), cardiovascular endurance (i.e., 6MWT score), muscular strength and endurance (i.e., isometric push-up, modified curl-up, and hand grip strength scores), and flexibility (i.e., back-saver sit-and-reach score). All statistical analyses were performed using SPSS (version 20.0; IBM Corp, Armonk, NY, USA), and a *p* of <0.05 was considered significant for all tests.

## 3. Results

The characteristics of the participants are described in Table 1. The sample consisted of 33 men and 27 women with mild to moderate ID, with an average age of 39.19 ± 11.70 years (range 19.20–70.20). The majority of the participants had moderate ID (*n* = 52, 87%), and approximately half had a BMI outside the normal range (*n* = 27, 45%).

Every participant completed the study. The overall average scores for each physical fitness component were as follows: BMI of 23.88 ± 3.58 kg/m^2^; 6MWT score of 390.08 ± 123.48 m; isometric push-up test time of 35.48 ± 11.64 s; 9.07 ± 10.92 curl-ups; right and left handgrip strength of 18.46 ± 12.78 and 18.14 ± 13.15 kg, respectively; and back-saver sit-and-reach distances of 14.03 ± 12.21 and 14.93 ± 11.81 cm for the right and left legs, respectively.

### 3.1. Gender, Age, and Intellectual Disability (ID) Level Differences in Physical Fitness Measures

Men scored significantly higher in the abdominal muscular endurance (*p* < 0.01) and grip strength (both hands; all *p* < 0.01) tests than women (Table 2). No significant differences in flexibility, cardiovascular endurance, upper-body muscular endurance, or BMI were observed between the genders.

Cardiovascular endurance, BMI, and right leg flexibility were significantly better (all *p* < 0.05) among younger adults (aged < 39 years) than among older adults (≥39 years; Table 2). No significant differences were observed in any other physical fitness measures. In addition, no differences were found in physical fitness measures, except BMI (*p* < 0.05), between participants with mild and moderate ID (Table 2).

### 3.2. Correlation and Regression Analyses of PA Levels

Table 3 presents the partial correlation coefficients between all PA variables (daily SA, LPA, and MVPA time and steps/day) and physical fitness scores in all participants. Low correlations were observed between SA time and abdominal muscular endurance and between LPA time and cardiovascular endurance, abdominal and upper-body muscular endurance, and flexibility in both legs. Low correlations were also observed between MVPA time and cardiovascular endurance, abdominal muscular endurance, grip strength (both hands), and left leg flexibility as well as between overall PA (steps/day) and abdominal and upper-body muscular endurance and grip strength (both hands). A moderate correlation was observed between overall PA (steps/day) and cardiovascular endurance. No significant correlations were discovered between any PA variables and BMI.

The stepwise multiple regression analysis indicated that MVPA was positively correlated with abdominal muscular strength and endurance, and SA was negatively correlated with abdominal muscular strength and endurance (Table 4). Both MVPA and SA were predictors of abdominal muscular strength and endurance. MVPA explained 15% of the variance in abdominal muscular strength and endurance (*R* = 0.39; *R*^2^ = 0.15; Δ*R*^2^ = 0.14, F(1, 58) = 10.54, *p* < 0.01). MVPA and SA together accounted for 23% of the variance in abdominal muscular strength and endurance (*R* = 0.48; *R*^2^ = 0.23; Δ*R*^2^ = 0.21, F(2, 57) = 8.60, *p* < 0.01).

In addition, MVPA was positively correlated with grip strength in both hands and explained 27% (*R* = 0.52; *R*^2^ = 0.27; Δ*R*^2^ = 0.25, F(1, 58) = 20.90, *p* < 0.01) and 25% (*R* = 0.50; *R*^2^ = 0.25; Δ*R*^2^ = 0.23, F(1, 58) = 18.97, *p* < 0.01) of the variance in the grip strength of the right and left hands, respectively. Furthermore, overall PA (steps/day) was positively correlated with cardiovascular endurance and explained 32% of the variance (*R* = 0.57; *R*^2^ = 0.32; Δ*R*^2^ = 0.31, F(1, 58) = 27.53, *p* < 0.01). Moreover, LPA was positively correlated with flexibility (both legs) and upper-body muscular endurance. LPA explained 13% (*R* = 0.36; *R*^2^ = 0.13; Δ*R*^2^ = 0.12, F(1, 58) = 8.67, *p* < 0.01) and 14% (*R* = 0.37; *R*^2^ = 0.14; Δ*R*^2^ = 0.13, F(1, 58) = 9.51, *p* < 0.01) of the variance in right and left leg flexibility, respectively. In addition, LPA explained 18% of the variance in upper-body muscular endurance (*R* = 0.42; *R*^2^ = 0.18; Δ*R*^2^ = 0.16, F(1, 58) = 12.27, *p* < 0.01).

## 4. Discussion

To the best of the authors’ knowledge, no published studies have investigated the correlations of individual and modifiable factors with physical fitness components in adults with ID. Three fixed individual characteristics were negatively associated with physical fitness among individuals with ID and must be considered in establishing reference values: old age, female gender, and mild disability. The associations of the modifiable PA factors with muscular strength and endurance, cardiorespiratory fitness, and flexibility indicate a potential path for improving physical fitness in this population.

### 4.1. Gender, Age, and ID Level Differences in Physical Fitness Measures

Male participants scored higher in abdominal muscular endurance and grip strength than female participants; this result echoes research that indicated men with ID had higher muscular strength and endurance [42]. However, previous research has indicated that women with ID have less cardiorespiratory endurance [5] and greater flexibility [42], but the current study observed no statistically significant gender differences in these parameters. No gender differences in BMI were observed either. The BMIs of women and men in this study were 23.19 and 23.83, respectively, both of which are within the normal range. This study does not support earlier findings of a high prevalence of obesity among individuals with ID [43].

The younger participants with ID had lower BMIs and greater cardiorespiratory endurance and greater flexibility compared to the older participants. These findings are similar to those of research indicating that age unfavorably affects the BMI [44], cardiovascular endurance, and flexibility of adults with ID [45]. However, no effect on muscular strength and endurance was observed in the present study, which contradicts a longitudinal study indicating a significant age-related decline in abdominal muscular strength and endurance over a period of 13 years in middle-aged adults (mean age 41.2 ± 9.72 years) with an ID [45]. Muscular strength and endurance decline more gradually with age than do other physical fitness performance variables [46]. Although this decrease in strength and endurance seems to be related to increasing age and muscle-mass loss, it is probably a consequence of the greater physical inactivity that comes with age.

No significant differences were observed in most physical fitness components between participants with mild and moderate ID. However, the mean BMI of the participants with mild ID was higher than that of the participants with moderate ID. This finding is unexpected because those with more severe ID have greater limitations in completing activities of daily living [47]; thus, less PA [48] and higher BMIs [20] are commonly associated with more severe ID. The difference in BMI between participants with mild and moderate ID in the present study remains unexplained. Caution should be exercised in the generalization of the current findings because this study was limited by a small overall sample and even smaller sample of participants with mild ID.

### 4.2. Correlation and Regression Analyses of PA Parameters

As in the general population, being physically active was positively associated with cardiorespiratory endurance, muscular strength and endurance, and flexibility in adults with ID, suggesting that these factors can be improved by increasing PA and decreasing SA. These results are in agreement with an intervention study in adults with ID that indicated positive effects of PA on several physical fitness outcomes [49]. Moreover, previous research has indicated that people with ID engage less frequently in PA [3], are less physically fit [5], live more sedentary lives [4], and are more likely to be overweight or obese [6] than the general population. Such infrequent PA and poor fitness suggest that any PA increase, regardless of duration or intensity, can increase the physical fitness of individuals with ID and improve their health [50].

In this study, cardiorespiratory endurance and muscular strength and endurance were positively and significantly related to the number of steps taken daily by adults with ID. This result is in agreement with a study that identified a positive relationship between PA (i.e., steps per day) and cardiorespiratory endurance in youths with moderate to severe ID [51]. In addition, regression modeling in the current study revealed that overall PA, as measured in steps per day, was a significant predictor of cardiorespiratory endurance in adults with ID. Walking is the most frequently proposed activity in PA programs for adults with ID and can improve cardiorespiratory fitness [52]. Furthermore, walking is often the primary PA engaged in by adults with ID [53] and involves minimal joint impact and fall risk. Therefore, walking should be considered for PA interventions targeting this population.

The present study demonstrated a close relationship between physical fitness components (i.e., cardiovascular endurance, muscular strength and endurance, and flexibility) and MVPA in adults with ID. Current global PA guidelines recommend only MVPA; however, the present results reveal a positive association of LPA with upper-body muscular endurance as well as flexibility, suggesting that any increase in PA (including LPA) can improve the physical fitness of adults with ID. Limited evidence of the health benefits of LPA is available [25], but the present findings provide new information regarding the associations of LPA and physical fitness in adults with ID. Examples of LPA include activities that range from no movement (e.g., standing) to movement with an intensity between 1.5 and 2.9 metabolic equivalents (e.g., brisk walking). Adults with ID may be more willing to replace SA with LPA than with MVPA because it can be more easily incorporated into their daily lives. LPA-based interventions may involve restructuring the work environment [54], such as by providing standing desks in the workplace or encouraging individuals to take the stairs instead of the elevator. Other interventions may involve promoting movement, such as by encouraging active computer gaming over sedentary TV viewing or 2-min bouts of standing or walking during regular daily activities at service provider facilities. Future PA guidelines may benefit from including recommendations for LPA in addition to MVPA. If the activity of adults with ID can be shifted from a pattern of predominant SA to one featuring more LPA, interventions targeting MVPA may become more successful because the transition from LPA to MVPA represents a more natural progression along the PA continuum. Furthermore, regression modeling revealed that LPA to be significantly associated with upper-body muscular endurance and flexibility in adults with ID. These findings support the notion that replacing some SA with LPA aids in the prevention of unfavorable metabolic outcomes in adults with and without disabilities [26,55].

In this study, BMI was not related to any PA variables. In the current study, 38% (41% of women and 36% of men) of the participants were classified as overweight or obese; this result is similar to those observed in adult Special Olympics participants with ID in the Asia–Pacific (40% of women and 37% of men) and East Asia (32% of women and 27% of men) regions [43]. Temple et al. [43] demonstrated that adult Special Olympics participants have high levels of overweight and obesity, particularly those who are women and those from North America (76% of women and 68% of men), Eurasia (57% of women and 48% of men), or Latin America (51% of women and 34% of men). These high figures are not specific to one regain but are consistent globally. Obesity is two to three times more prevalent among individuals with ID because of obesogenic medication and sedentary lifestyles [56]. However, in the study of Temple et al. [51], participation in physical exercise did not protect such adults from obesity, which remains the largest health concern among this population. Other factors associated with overweight and obesity in individuals with ID include nutritional habits, psychotropic medication, altered metabolic rates [57], and Down’s syndrome [58]. Chronic inflammation may also be an underlying pathological condition when people become obese [59]. Therefore, the mechanisms of the exercise effectiveness such as prevention of chronic inflammation and oxidative stress cannot be overlooked. Nevertheless, few PA interventions have resulted in weight loss in youths [60] or adults with ID [61], suggesting no salient relationship between PA and BMI in this population. Thus, more studies on the impact of PA on the body composition of adults with ID are required [52].

Several limitations of this study should be noted. First, the sample sizes were small; thus, the current findings should be generalized with caution. Second, this was a cross-sectional study; therefore, causal relationships could not be established. Third, including different numbers of individuals with each ID level limited the validity of the comparisons. Fourth, the tests used to measure fitness components may be insensitive to gender, age, and ID level, and such insensitivity may invalidate conclusions that these factors affect physical fitness. Fifth, there is no comparison group; therefore, it cannot determine if the fitness decline is due to the ID, aging, or inactivity. Finally, the use of the PA and fitness measurement methods permitted limited assessment of other PA and fitness functions.

In conclusion, in addition to MVPA, LPA may increase physical fitness in adults with ID. The current findings suggest the importance of PA and various PA intensities to physical fitness; moreover, LPA may be as beneficial as MVPA to the physical fitness of this population. Future studies should focus on the development of effective PA interventions for adults with ID by comparing MVPA and LPA. Furthermore, special attention should be paid to the physical fitness of women with ID and older adults with ID. Such research may highlight health inequalities and, therefore, be useful for planning and evaluating PA interventions. Representative samples are essential for examining PA intensity and the fitness levels of adults with mild to profound ID.

## Figures and Tables

**Table 1:** Participants’ characteristics for the total group.

	*N*	%	*M*	*SD*
*N*	60		--	--
Gender				
Males	33	55	--	--
Females	27	45	--	--
Age (years)			39.19	11.70
<39 years	30	50	29.80	5.59
≥39 years	30	50	48.57	8.08
ID levels				
Mild	8	13	--	--
Moderate	52	87	--	--
BMI categories				
Underweight	4	7	17.93	0.56
Normal	33	55	22.32	2.48
Overweight	21	35	26.72	1.37
Obese	2	3	31.82	0.98
PA levels				
SAmin	--	--	517.69	103.19
LPAmin	--	--	275.25	76.86
MVPAmin	--	--	16.67	13.84
Steps	--	--	6486	2935

*Note.* ID = intellectual disability; BMI = body mass index; PA = physical activity; SAmin = sedentary activity time (min/day); LPAmin = light physical activity time (min/day); MVPAmin = moderate-to-vigorous physical activity time (min/day).

**Table 2:** Average score on the physical fitness measures of participants with ID by gender, age and ID level (*n* = 60).

Variables	By Gender			By Age-Group			By ID-Level		
	Males	Females		<39 Years Old	≥39 Years Old		Mild	Moderate	
	*n* = 33	*n* = 27	*p*_1_	*n* = 30	*n* = 30	*p*_2_	*n* = 8	*n* = 52	*p*_3_
BMI (kg/m^2^)	23.83 ± 4.12	23.19 ± 4.58	0.829	22.03 ± 4.91	25.04 ± 2.99	**0.007**	26.54 ± 3.51	23.08 ± 4.26	**0.018**
6-min walk test (m)	409.55 ± 115.60	366.30 ± 130.71	0.389	429.17 ± 126.10	351.00 ± 109.36	**0.017**	417.50 ± 134.54	385.87 ± 122.55	0.249
Isometric push-up (s.)	36.15 ± 10.62	34.67 ± 12.94	0.689	36.07 ± 10.61	34.90 ± 12.74	0.936	35.00 ± 14.14	35.56 ± 11.37	0.801
Modified curl-up (n)	12.33 ± 11.66	5.07 ± 8.57	**0.001**	10.50 ± 9.85	7.63 ± 11.89	0.083	12.13 ± 11.56	8.60 ± 10.86	0.343
Grip strength (kg)									
Right hand	23.53 ± 14.08	12.26 ± 7.36	**0** **.000**	19.15 ± 10.37	17.77 ± 14.96	0.446	18.56 ± 9.26	18.44 ± 13.31	0.486
Left hand	22.82 ± 14.84	12.43 ± 7.72	**0.002**	19.38 ± 10.72	16.90 ± 15.28	0.231	18.13 ± 8.75	18.14 ± 13.76	0.655
Sit-and-reach (cm)									
Right leg	12.11 ± 13.21	16.39 ± 10.63	0.129	17.52 ± 12.37	10.55 ± 11.19	**0.028**	12.19 ± 13.61	14.32 ± 12.10	0.439
Left leg	12.92 ± 12.80	17.37 ± 10.17	0.103	17.00 ± 11.85	12.85 ± 11.59	0.133	12.19 ± 13.61	14.32 ± 12.10	0.613

*Note.* ID = intellectual disability; BMI = body mass index; *p*_1_ = Difference between gender based on the nonparametric Mann–Whitney *U* tests. *p*_2_ = Difference between age-group based on the nonparametric Mann–Whitney *U* tests. *p*_3_ = Difference between ID-level based on the nonparametric Mann–Whitney *U* tests. Statistically significant values are shown in bold (*p* < 0.05).

**Table 3:** Partial correlation coefficients between physical fitness outcomes, physical activity, and sedentary activity time in participants with ID (*n* = 60).

	SAmin		LPAmin		MVPAmin		Steps	
	*r*	*p*	*r*	*p*	*r*	*p*	*r*	*p*
BMI (kg/m^2^)	0.10	0.874	−0.01	0.579	−0.04	0.210	−0.07	0.169
6-min walk test (m)	−0.11	0.438	0.40 **	0.002	0.36 **	0.006	0.51 **	0.000
Isometric push-up (s.)	−0.10	0.470	0.43 **	0.001	0.14	0.306	0.32 *	0.017
Modified curl-up (n)	−0.31 *	0.018	0.33 *	0.012	0.31 *	0.019	0.28 *	0.034
Grip strength (kg)								
Right hand	0.04	0.743	0.22	0.096	0.43 **	0.001	0.30 *	0.022
Left hand	0.03	0.820	0.25	0.062	0.41 **	0.002	0.31 *	0.018
Sit-and-reach (cm)								
Right leg	−0.15	0.279	0.31 *	0.017	0.26	0.050	0.21	0.117
Left leg	−0.13	0.339	0.36 **	0.006	0.31 *	0.017	0.26	0.054

*Note.* ID = intellectual disability; BMI = body mass index; SAmin = sedentary activity time (min/day); LPAmin = light physical activity time (min/day); MVPAmin = moderate-to-vigorous physical activity time (min/day); The correlation is graded as: (a) 0 to 0.25 = little; (b) 0.26 to 0.49 = low; (c) 0.50 to 0.69 = moderate; (d) 0.70 to 0.89 = high; and (e) 0.90 to 1 = very high [41]; level of significance * *p* < 0.05, ** *p* < 0.01.

**Table 4:** Stepwise multiple regression analysis results for adults with ID (*n* = 60).

Dependent	Independent	B [95%CI]	*SE*	*β*	*t*	*p*
Modified curl-up	SAmin	−0.03 [−0.06, −0.01]	0.01	−0.28	−2.41	0.019
	MVPAmin	0.31 [0.12, 0.50]	0.10	0.39	3.25	0.000
6-min walk test	Steps	0.02 [0.02, 0.03]	0.01	0.57	5.25	0.000
Isometric push-up	LPAmin	0.06 [0.03, 0.10]	0.02	0.42	3.50	0.000
Right hand grip strength	MVPAmin	0.48 [0.27, 0.68]	0.10	0.52	4.57	0.000
Left hand grip strength	MVPAmin	0.47 [0.26, 0.69]	0.11	0.50	4.36	0.000
Right leg sit-and-reach	LPAmin	0.06 [0.02, 0.10]	0.02	0.36	2.94	0.005
Left leg sit-and-reach	LPAmin	0.06 [0.02, 0.10]	0.02	0.38	3.08	0.003

*Note.* ID = intellectual disability; CI = confidence interval; SAmin = sedentary activity time (min/day); LPAmin = light physical activity time (min/day); MVPAmin = moderate-to-vigorous physical activity time (min/day). Stepwise criteria: probability-of-F-to enter ≤0.05, probability-of-F-to remove ≥0.10.

## Data Availability

Not applicable.
